# Association of Mitochondrial DNA Haplogroups with Pediatric Systemic Lupus Erythematosus Disease Activity, Damage Scores, and Lupus Nephritis

**DOI:** 10.3390/jcm15010086

**Published:** 2025-12-23

**Authors:** Viraat Udar, Shari R. Atilano, Alexis V. Stephens, Ryan Yu-Sheng Chang, Nicholas J. Jackson, Steven Y. Chang, Marilyn Chwa, Deborah McCurdy

**Affiliations:** 1 Genomeadvisors Inc., La Mirada, CA 90638, USA; viraatudar9@gmail.com; 2 Gavin Herbert Eye Institute, University of California Irvine, Irvine, CA 92697, USA; satilano@hs.uci.edu (S.R.A.); rychang1@uci.edu (R.Y.-S.C.); sychang2@hs.uci.edu (S.Y.C.);; 3 Department of Pediatric Rheumatology, University of California Los Angeles, Los Angeles, CA 90095, USA; avstephens@mednet.ucla.edu; 4 Department of Medicine Statistics Core, University of California, Los Angeles, CA 90095, USA; njjackson@mednet.ucla.edu

**Keywords:** systemic lupus erythematosus (SLE), pediatric SLE (pSLE), mitochondria, mitochondrial (mt) DNA haplogroup, systemic lupus erythematosus Disease Activity Index (SLEDAI-2K), Systemic Lupus Internal Collaborating Clinics (SLICC) Damage Index (SDI), lupus nephritis

## Abstract

Mitochondria, which have critical roles in energy metabolism and oxidative regulation, also have a role in immune regulation including T cell activation, NET formation, inflammation, and apoptosis. More than 50% of those with systemic lupus erythematosus (SLE) have lupus nephritis due to kidney damage from immune complex deposition. Disease severity is reported to be greater in certain lineages. Mitochondrial DNA (mtDNA) haplogroups, which reflect maternal lineages, may modulate immune balance and disease outcomes in SLE. **Methods:** DNA was extracted from 25 consecutive, consenting pediatric patients that fulfilled the 1997 criteria for SLE and their maternal mitochondrial DNA (mtDNA) haplogroups were determined through next-generation sequencing (NGS). **Results:** This study evaluated the associations between mtDNA haplogroups, lupus nephritis, and organ damage in four mtDNA haplogroups: African (n = 5), Amerindian (n = 12), Asian (n = 4), and Caucasian (n = 4). Clinical data, SLE Disease Activity Index (SLEDAI-2K), SLICC Damage Index (SDI), and renal biopsy findings were analyzed. Median SLEDAI-2K scores were higher in Amerindian (10) and African (8) patients than in the Caucasian (5.5) and Asian (3) groups, with significant differences between Amerindian vs. Caucasian (*p* = 0.045) and Amerindian vs. Asian (*p* = 0.008). Irreversible organ damage (SDI > 1) was more frequent in Amerindian (54%) and African (40%) patients. Lupus nephritis occurred most often and most severely (Class III–IV, CKD) in the Amerindian (85%) and African (80%) groups, while Caucasian and Asian patients more often showed milder, membranous disease without CKD. Conclusion: Although limited by the small sample size, pediatric SLE severity and renal involvement were found to be greater in Amerindian and African mtDNA haplogroups, suggesting that mitochondrial lineage may contribute to ethnic disparities in SLE.

## 1. Introduction

Systemic lupus erythematosus (SLE) is a chronic autoimmune disease in which most patients have six or more autoantibodies directed against sequestered antigens, such as nuclear and cytoplasmic targets, but also against membrane proteins and, as noted more recently, against mitochondria from damaged cells. Ongoing immune dysregulations result in the subsequent development of immune complexes and autoreactive T cells, resulting in chronic inflammation and organ damage. SLE is a devastating life-changing disease that primarily affects young women in their childbearing years [1,2,3,4]. Though more common in adults, 20% of patients with SLE are diagnosed during childhood, most often at the onset of puberty, and it is estimated to affect 3.3–8.8/100,000 children. Though the skin and musculoskeletal system are almost universally involved, the kidney is the major organ that is most often affected by inflammatory damage in pediatric SLE (pSLE) with over 60% developing lupus nephritis and up to 15% progressing to end-stage renal disease and dialysis. Approximately 20–30% of patients with pSLE have central nervous system involvement and may have headaches, cognitive changes, and seizures [1,2,3,4]. Any of the other organs can also be involved.

Because of the protean manifestations and variable courses, criteria have been established to confirm the diagnosis and tools have been created to determine disease activity and organ damage. The Systemic Lupus Erythematosus Index 2000 (SLEDAI-2K) is a tool used to measure persistent active or new onset disease activity over the last 30 days [5]. The SLEDAI-2K assesses 16 clinical parameters and 8 laboratory values; the scores range from 0 to 105, with scores 0-4 with little disease activity, and >4 indicating active disease. This study used scored higher than 6 indicating active disease and a need for therapy. This score varies with time and therapy; however, it is useful for adjusting treatment and if it consistently high, it is concerning as a harbinger of organ damage. The Systemic Lupus International Collaborating Clinics Damage Index (SDI) was developed to quantify damage over the course of the disease and has been shown to be able to detect irreversible damage that correlates with mortality [6]. A positive score indicates organ damage that affects quality of life. Kidney involvement is a major cause of morbidity and the most common cause of death in SLE patients [7]. Links between mitochondria and immune regulation and stimulation are increasingly recognized [8]. Wang et al. established a link between mtDNA in NETs, anti-mtDNA antibodies, and plasma dendritic cell (PDC) IFNα pathogenesis in SLE [9].

There are many factors that contribute to the pathogenesis of SLE. The risk in certain populations suggests that there are genetic factors related to ancestral descent. Most genetic studies have concentrated on nuclear genes and genome-wide association studies (GWASs) have identified multiple candidate genes [10]. TLR7, a sensor of viral RNA that binds to guanosine has been implicated in the pathogenesis of SLE [11]. Brown et al. have described human SLE caused by a gain-of-function variant of the nuclear gene TLR7 [12]. However, mtDNA genetics have not been well studied in SLE. Altered reactive oxygen species (ROS) metabolism in SLE has been reported for decades, with increased ROS levels associated with increased inflammatory damage [13,14,15,16].

**Figure 1:**
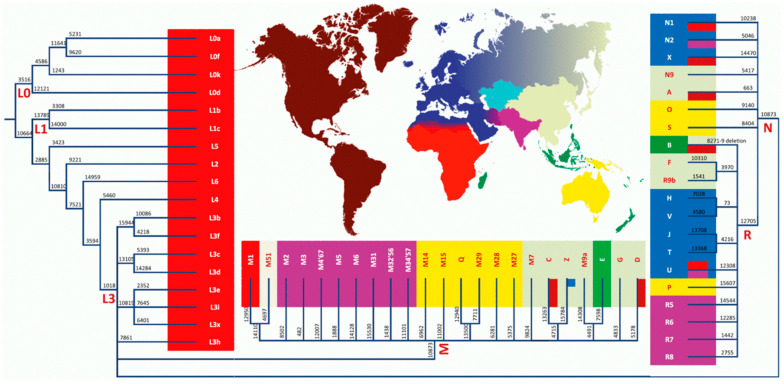
Global haplogroup distribution. Figure adapted from Toomas Kivislid, “Maternal ancestry and population history from whole mitochondrial genomes” *Investigative Genetics* (2015). Licensed under CC by 2.0 (https://creativecommons.org/licenses/by/2.0, accessed 20 October 2023). Each letter represents a major mitochondrial haplogroup. The red L haplogroups are the earliest human mtDNA lineages. The numbers along the branches represent mutation positions that correspond to specific nucleotide changes in mtDNA. Haplogroup M and its derivatives (purple, yellow, light green) are seen in Asia; R subgroups appear in India (purple) and Europe (dark blue); N is seen in indigenous peoples in Australia and Asia, Indigenous people of the Americas predominately carry haplogroups A–D.

Mitochondria have their own DNA (mtDNA) which is inherited through the maternal lineage and is distinct from nuclear DNA. The human mtDNA has 16,569 nucleotide pairs, and codes for 37 genes including 13 protein subunits essential for oxidative phosphorylation (OXPHOS), 2 ribosomal RNAs, and 22 transfer RNAs [17,18,19]. Most mitochondrial proteins (~1500–2000) are encoded by nuclear DNA and are imported into the mitochondria where they participate in energy production and genome stability and are reported to alter the immune responses [17,18,19,20,21]. As human populations migrated over the millennia, single-nucleotide polymorphisms (SNPs) accumulated in human mtDNA, forming unique haplogroups that can be used to identify African, Middle Eastern, European, Asian, Amerindian (indigenous in the Americas; referred to as Hispanic or LatinX), and Australian populations (Figure 1) [22]. Each mtDNA haplogroup appears to have a metabolic benefit for survival in its specific environment [23,24,25]. Although it has long been recognized that mitochondria are associated with metabolism and are the “powerhouses” of cellular metabolism, there is increasing appreciation of the effects of mitochondria on immune regulation [26,27,28] and their contribution to disease states [29]. The effects on immune regulation may be a result of different metabolisms in the mtDNA haplogroups or there may be a direct effect on immune regulation through interactions with cellular DNA, both nuclear and cytoplasmic, in specific populations [30,31]. Although there is no clear association between mtDNA haplogroups and autoimmune disease, there is literature supporting mitochondrial effects on the immune system. For example, gene deficiencies in the electron transport chain (ETC) have been shown to be closely related to the development of kidney disease, providing evidence that mitochondrial integrity is a key player in the early development of CKD [32].

The association of mtDNA haplogroups, which are found in distinct ethnicities, with the pathogenesis of SLE is supported by the following two facts: (1) specific ethnic populations are more susceptible to SLE and (2) tissues with high energy demands (kidney, neurological, cardiac, ocular, and musculoskeletal tissues) are significantly affected in SLE. The focus of this study is to determine if different mitochondrial DNA haplogroups are associated with an increased risk of lupus disease activity, organ damage, and/or lupus nephritis.

## 2. Materials and Methods

### 2.1. Epidemiology and Clinical History of Pediatric Patients with SLE

Approximately 25% of patients are diagnosed with lupus before 20 years old, with the majority of diagnoses occurring at puberty. Our study design involved recruiting patients with pediatric SLE (pSLE) whose disease started at 18 years or younger (childhood). The children attended the Pediatric Rheumatology Lupus Clinic at UCLA and were diagnosed with SLE based on the 1997 American College of Rheumatology (ACR) criteria [1]; all had 4 or more criteria. The 1997 ACR criteria for a diagnosis of SLE include a malar rash that goes over the bridge of the nose or a discoid rash that is scaley and well demarcated; photosensitivity; oral or nasal ulcers; non-erosive arthritis involving 2 or more peripheral joints; serositis of the lungs or pericardium; proteinuria of >500 mg/24 h or cellular casts; seizures, psychosis, or delirium; autoimmune hemolytic anemia, thrombocytopenia, or leukopenia; and positive laboratory tests for antinuclear antibody (ANA), anti-ds-DNA, anti-Sm, and/or antiphospholipid antibodies (Table S1 in the Supplemental Materials). Consecutive patients and their parents were asked to participate in the study at the time of a necessary blood draw. Their patients or guardians read and signed the informed consent form and the children over 12 years old signed an assent form from the Institutional Review Board of the University of California, UCLA (IRB #19-001470). All clinical investigations and protocols were conducted according to the principles of the Declaration of Helsinki and approved by the appropriate investigational review boards (University of California, Los Angeles, CA, USA). The patients were enrolled at a clinic visit. The clinic is based in Los Angeles, California, so ethnicity bias based on location cannot be ruled out. The patients were being followed to monitor disease activity at the time of the study. Their disease activity was determined using the SLE Disease Activity Index (SLEDAI-2K), which was performed after the clinical and laboratory assessments at the end of the visit. SLEDAI-2K scores range from 0 to 29, with >6 considered active. All patients were on therapy with a combination of prednisone and hydroxychloroquine and, in some cases, mycophenolate mofetil. All patients were monitored for renal disease and if there was persistent proteinuria, hematuria, casts, or an elevated creatinine, a renal biopsy was performed. The biopsy results in their charts were reviewed in a retrospective manner. Renal pathologists had analyzed the biopsy for activity and chronicity following the World Health Organization (WHO) criteria. Lupus nephritis was classified using the WHO criteria: Class I (normal on light microscopy), Class II (mesangial proliferation (Mes)), Class III (Focal Proliferative Glomerulonephritis (FPGN)), Class IV (Diffuse Proliferative Glomerulonephritis (DPGN)), and Class V (membranous glomerulonephritis (Mem)). Renal damage was classified as chronic kidney damage (CKD) or end-stage renal disease (ESRD). For the purposes of this study, the Systemic Lupus International Collaborating Clinics (SLICC) Damage Index (SDI) was also used to determine irreversible damage in each mtDNA haplogroup. The SDI was assessed from the charts in a retrospective manner. All patients were followed for at least 1 year and up to a period of 16 years prior to being enrolled in the study.

### 2.2. DNA Extraction

DNA was extracted from the blood drawn in sodium citrate vials from the pSLE patient during a routine blood draw using a kit (DNeasy Blood and Tissue Kit, Qiagen, Germantow MD, USA).

### 2.3. Next-Generation Sequencing Method

DNA extracted from blood was utilized for next-generation sequencing (NGS) [33,34]. Targeted primers spanning the entire mitochondrial genome were used for the library preparation using the TruSeq Amplicon kit (Illumina, San Diego, CA, USA). The library preparation and sequencing were conducted as described previously [33]. NGS technology allows for independent sequencing of both strands of mtDNA in both directions, resulting in higher sensitivity and specificity; it was used for haplogrouping. The minimum read depth of the sequencing was 1000×, the average was 154,562×, and the maximum was 684,346×. Phylotree 17 was used to analyze the mtDNA haplogroups [34]. The Amerindian haplogroups were defined as haplogroups A, B, C, and D, with further splits based on published data. The Caucasian lineage was defined as H, J, T, V, U, K, W, X, and I. Variants with an minor allele frequency (MAF) between 80 and 100% were classified as homozygous. Variants with an MAF between 30 and 80% were classified as heterozygous.

### 2.4. Statistics

Between-mtDNA haplogroup differences in demographic and clinical characteristics were assessed using a one-way analysis of variance for continuous variables and the exact test for categorical variables (SLEDAI-2K and SDI scores). They were summarized for each mtDNA haplogroup using medians with the 25th and 75th percentiles reported. Between-group differences in SLEDAI-2K and SDI scores were assessed using a Kruskal–Wallis test with post hoc pairwise comparisons conducted using a Wilcoxon Rank-Sum test. Due to the SDI having a predominance of scores equal to zero, we additionally assessed the SDI scores as a binary variable (zero vs. non-zero scores). Between-group differences for this binary variable were assessed using an exact test. Lastly, associations between disease duration and disease activity measures were examined using Spearman correlations. All analyses were conducted in Stata version 18 (Stata Corp LLC, College Station, TX, USA) and a two-sided *p* value less than 0.05 was considered statistically significant.

## 3. Results

### 3.1. Baseline Demographic Age

Information regarding sex and SLE duration was collected (Table 1). The SLEDAI-2K was used to determine disease activity, and the SDI was used to determine irreversible organ damage at the time of the sample acquisition. Based on mtDNA NGS analysis, the mtDNA haplogroups of all of the samples were determined and they were assigned to four key maternal lineages: African, Amerindian (often referred to as Hispanic or LatinX), Caucasian, and Asian. There were 5 African patients with haplogroups (L1c2b1b, L1c3, and L3) that are predominantly found in African maternal lineages; 12 Amerindian patients with haplogroups (A2u, A2j, A2, A2h1, D1c, A2o, A2w, C1c*4, C1b, and B2d haplogroups) that are predominantly found in North, Central, and South American maternal lineages; 4 Caucasian patients had haplogroups (H1aq1, U5a1d1, H3, T2b21) that are predominantly found in Caucasian maternal lineages; and 4 Asian patients had haplogroups (B4c1b2a, N, D4, and D5c2) that are predominantly found in east Asian maternal lineages. Of note, ethnic self-identity did not always correlate with the maternal mtDNA haplogroup. There was one patient in the African mtDNA haplogroups and three in the Caucasian mtDNA haplogroups that self-identified as Hispanic.

### 3.2. Age and Disease Duration

A comparison of the baseline demographics of the four maternal lineages (African, Amerindians, Caucasian, and Asian) is shown in Table 2. There was no difference in the sex ratios between the four mtDNA haplogroups. However, the study population was predominantly female (85% vs. 15%). Although it is known that females have a higher risk, our study population did not demonstrate a significant bias despite the younger age and smaller sample size. Statistical analysis revealed no significant difference (*p* = 0.99) in the distribution of females within the four haplogroups. The age of the patients in the four groups (African, Amerindian, Caucasian, and Asian) was similar, with an average age of 15.6 years and a range of 8 to 23 years old. The average age at diagnosis was 10.5 years and ranged from 2 to 18 years. The disease duration was also similar in all four groups, with an average of 5.2 years and a range of 0.25 to 17 years, underscoring the chronic nature of pSLE and the need for ongoing care.

### 3.3. Correlating Disease with mtDNA Haplogroup

Statistical analysis was carried out to compare the SLEDAI-2K [33] and SDI [34] scores of the different haplogroups. The comparison of the SLEDAI-2K of the four maternal lineages indicated a higher median score in the Amerindian (Score ave = 11.7) and African Score ave = 7.2) maternal lineages compared to the Caucasian (Score ave = 4.75) and Asian (Score ave = 3) maternal lineages (Table 3). Similar observations were also found for non-zero SDI scores, with higher percentages (54% and 40%) in the Amerindian and African maternal lineages. When individual maternal lineages were compared to each other, a significant omnibus *p* value of 0.017 was obtained. Significant differences in SLEDAI-2K scores were only observed between the Amerindian vs. Caucasian () and the Amerindian vs. Asian (*p* = 0.008) maternal lineages. However, there was no significant difference between Amerindians vs. Africans and Caucasian vs. Asian haplogroups. No significant differences were observed for the SDI scores when individual maternal lineages were compared to each other. Though the SLEDAI 2K varies with disease exacerbation and remission and therapies, those with more consistently high levels over time are at more risk for poor outcomes. Although the SLEDAI 2K was assessed at a single time point, certain mtDNA haplogroups had significantly higher numbers at the time of blood collection, which occurred at different times in the disease course of the patients (i.e., some were collected shortly after diagnosis, and some collected several years after diagnosis).

The SDI scores were examined in two ways: (1) treating SDI as a continuum and (2) examining the proportion of non-zero SDI scores across groups. An SDI score > 0 indicates organ damage and was noted in 9/27 (33%) patients in the overall sample, with 0% in those of Asian and Caucasian ancestry and 54% and 40% in those of Amerindian and African ancestry, respectively (Table 3). While omnibus tests indicated no overall differences in continuous SDI (*p* = 0.12) or proportion with a non-zero SDI (*p* = 0.10), the high SDI in the African and Amerindian groups suggests that these mtDNA haplogroups are at greater risk for irreversible damage. Our study did not take into account differences in economic or psychosocial factors related to the disease. These results demonstrate a higher disease severity in the Amerindian maternal lineage compared to the Caucasian and Asian maternal lineages.

### 3.4. Associations of Disease Duration with Disease Activity

The study compared the patients with SLEDAI-2K and SDI scores > 1 among the different mtDNA haplogroups (Table 4). SLEDAI 2K scores were higher (greater than or equal to 6) in the Amerindian (92%) and African (80%) mtDNA haplogroups compared to the Asian (none) mtDNA haplogroup. Likewise, the SDI > 1 scores indicating irreversible damage showed a trend (*p* = 0.10) towards being higher in the Amerindian (54%) mtDNA haplogroups compared to the Caucasian and Asian mtDNA haplogroups, despite similar average ages and disease durations. The mean follow-up periods were 5 years for African, 4.8 years for Amerindian, 4.6 years for Caucasian, and 7.5 years for Asian patients.

The SLEDAI-2K scores indicate the severity of disease activity over the month prior to assessment. Higher SLEDAI-2K scores, indicating active disease (>6), were observed in the African (Score ave = 7.2) and Amerindian (11.7) mtDNA haplogroups compared to the Caucasian (4.7) and Asian (3) mtDNA haplogroups. This suggests that African and Amerindian patients experience more active disease for longer time periods and potentially acquire greater end organ damage despite similar or shorter follow-up durations.

### 3.5. Lupus Nephritis

We compared the incidence and classification of lupus nephritis of the pSLE patients in the four maternal lineages (Table 5). Class II lupus nephritis (LN) or mesangial LN, which has pathologically varying glomerular immune complex patterns that include predominantly mesangial deposits, was significantly more common in the Caucasian (25%) and Asian (50%) haplogroups compared to the Amerindian (0%) and African (0%) haplogroups. Lupus nephritis Class III/IV or focal and diffuse proliferative LN has pathological mesangial and subendothelial deposits with endocapillary hypercellularity and infiltration of inflammatory cells, often with fibrinoid necrosis and crescentic lesions [7]. These classes were significantly more common in the Amerindian (85%) and African (50%) haplogroups compared to the Asian (0%) maternal lineage. Lupus nephritis Class V or membranous patients have antibodies to podocytes and pathological subendothelial deposits with prominent duplication of glomerular basement membranes (GBMs). This class was noted to be more common in the Asian (50%) and Caucasian (25%) haplogroups compared to the Amerindian (7%) and African maternal lineages (0%). It is intriguing that the pathophysiology of Classes II and V was more commonly observed in the Asian maternal lineage and Class III/IV was more common in the Amerindian and African maternal lineage. The incidence of CKD/ESRD was significantly higher in the Amerindian (15%) and African (20%) maternal lineages compared to the Caucasian (0%) and Asian (0%) maternal lineages.

These results show trends, but the small sample size must be takien into account; associations are suggested, but not established. 

## 4. Discussion

Because of the small sample size, this is an exploratory, hypothesis-generating study that suggests a plausible association of the mtDNA haplogroups of Amerindian and African populations with the increased risk of renal disease compared to Caucasian and Asian populations. There is no clear association with the disease activity score, based on the SLEDAI-2K; but, since this tool only reflects the diseases activity for the preceding 30 days, the score changes as patients flare and are treated. Thus, although the differences in the SLEDAI-2K between the groups is interesting, it is not a reliable method for predicting outcomes. In fact, as the patients were treated, the SLEDAI-2K decreased, even as renal disease was detected and treated. Although a larger population of patients needs to be analyzed, these associations give credence to the role of mitochondria in immune dysregulation. Other studies have also shown an association of SLE with mitochondrial variants but they did not look at the same populations as in this study [30,31].

This study identified trends that indicate that Amerindian and African maternal ancestral origin populations (based upon their mtDNA haplogroup profile) are at a higher risk for SLE and lupus nephritis. However, the interpretation of these results should be taken with caution given that this study used a small data set and had low statistical power. A larger data set is needed for confirmation of our results. This suggests that there could be differences in SLE disease presentation and severity based on the mtDNA haplogroup as it affects metabolism and immune regulation. It is now recognized that mtDNA haplogroups differ in bioenergetics. Mitochondrial haplogroups have been reported to play a role in disease due to ROS production and mitochondrial activation of apoptosis [26]. With more ROS production, there is a predisposition to aging, neurodegenerative diseases, and cancer. In combination with environmental factors, mtDNA haplogroups may weigh heavily in the predisposition for certain diseases due to effects on energy production, ROS, and apoptosis [35,36,37,38].

It is interesting to note that there is a distinct difference in the LN classification within these four mtDNA haplogroups and may indicate a difference in etiology/pathogenesis. To be clear, it is difficult to address the correction for multiple comparisons because our sample size is small, but there is a suggestion that Class III and Class IV (proliferative glomerulonephritis) may have a different immune pathway than Class V (membranous glomerulonephritis). How the mitochondrial DNA in each distinct mtDNA haplogroup influences these changes remains to be determined. Studies suggest that the differences in mitochondrial DNA variations influence metabolism, favoring tolerance based on mitochondrial metabolism as opposed to a pro-inflammatory picture based on nuclear glycolysis. Or there may be specific cellular signaling pathways that include the expression of nuclear genes. In this study, each maternal lineage had distinct pathologies and therefore may indicate overlapping but different metabolism and immune responses, or molecular mechanisms related to neoantigens based on variants in the mtDNA. Other studies have shown that the C5a-C5aR1 axis promotes podocyte injury by enhancing Drp1-mediated mitochondrial fission, which could have significant implications for the treatment of LN [39].

The role of mitochondria in the regulation of the immune system has been increasingly recognized in the last decade. Mitochondria influence both the innate and acquired immune system through their metabolism, reactive oxygen species (ROS) production, and regulation of apoptosis [40,41]. As part of the innate immune system, mitochondria are involved in pathways that induce cytokines involved in antiviral and antitumor defenses. For example, cytosolic sensors can detect viral RNA and activate a protein, MAVS, on the outer mitochondrial membrane that induces Type I interferon (INF) production, which is essential in the immune response to viral infections [42]. As a byproduct of oxidative phosphorylation, ROS act as signaling molecules that activate pathogen killing, activate the inflammasome, and stimulate cytokine production. The stronger immune defense can, if the mitochondrial response is too robust, result in damaging inflammation. This occurs when ROS levels are high, as is seen in several autoimmune diseases. In addition, under metabolic stress, the mitochondria release mtDNA that is similar to bacterial DNA and activates immune sensors such as cGAS-STING and TLR9 that trigger an inflammatory response [41,43,44].

Cytokines, which are involved in inflammation, are often increased in SLE, especially Type I INF. The Type I INF signature, in which genes downstream of Type I INF are activated, is strong at the time of disease onset in SLE and may have been increasing for up to two years before the disease manifests [45]. This is partially genetically mediated and several genetic variations have been identified that increase Type 1 INF but more recent studies suggest that the mitochondria are also involved. Targeting Type I INF to decrease its activity is now used therapeutically in certain patients with SLE. 

Metabolism affects the acquired immune system and fuels T cell growth, migration, and immune function [46]. Glycolysis occurs in the cytoplasm of a cell, breaking down glucose into pyruvate and does not require oxygen; it can occur in anaerobic conditions. Effector T cells depend on glycolysis. T regulatory cells (Tregs) also rely on glycolysis for growth and migration, but require mitochondrial metabolism, through oxidative phosphorylation and fatty acid oxidation, for their immunosuppressive function [26]. Without modulation of T cell immune function by Tregs, there is loss of tolerance and autoimmune disease can develop. 

There are several limitations to this study; the most obvious and concerning is the interpretation of the data. As mentioned previously, the sample size is small, limiting the statistical significance and can only suggest trends rather than associations. An extended study is warranted and is underway. The second limitation is that the study did not address the psychosocial and economic factors that confound data interpretation and medical outcomes. All of the patients seen in the Lupus Clinic have insurance, either private or state funded. The access to medical care and medication is uniform, but issues such as the understanding of the SLE disease process and medical compliance, as well as parental consent to therapeutic regimens and access to transportation to medical appointments makes certain groups at risk for poor outcomes. The data to analyze these factors was not collected for analysis.

Although preliminary haplogroup-level association with SLE is discussed, penetrance was not accessed since the manifestation was not congenital. The lack of gene expression data limits the interpretation of which haplogroup variants could be influencing the phenotypic presentations of SLE. There is a lack of genomic DNA variant data and therefore, a meaningful interpretation of co-segregation of variants in both genomic DNA and mitochondrial DNA cannot be determined. Mitochondrial copy number variants were not assessed in this study and could potentially influence the disease phenotype, as has been observed by Liu et al. [47]. Additionally, cybrid-based studies using patient-acquired mitochondria can also help in the future, identifying the metabolic pathways that are influenced by these haplogroups.

Our population included mostly those of Amerindian descent, but those of African, Asian, and Caucasian descent were also included. It should be noted that patients often self-identify as being from one background but mtDNA analyses indicate a different maternal lineage than expected; it is therefore important to determine the mtDNA haplogroup maternal lineage to fully evaluate the role of mitochondria in pSLE. The next steps are to determine the association of pSLE with specific mtDNA variants (both germline and somatic) and to study the bioenergetics of pSLE patients’ mitochondria.

## Figures and Tables

**Table 1:** Baseline demographic, disease characteristics, and haplogroups of pSLE patients.

Number/Haplogroup Identity	Age at Dx	Age	SLE Duration	Sex	Haplogroup	SLEDAI 2K	SDI	Renal Bx WHO Class
African								
20–03	9	15	6	F	L1c2b1b	8	2	DPGN IV
20–16	18	19	1	M	L1c3	10	3	DPGN IV ESRD
20–19	10	12	2	F	L3	8	0	FPGN III
20–23	6	21	15	F	L3	2	0	DPGN IV
21–55	7	8	1	F	L3	8	0	No bx
Amerindian								
20–05	11	17	6	M	A2u	14	5	DPGN IV/CKD
20–07	16	18	2	F	A2j	16	2	Mem V
20–09	8	14	6	M	A2	18	1	FPGN III
20–11	11	12	1.5	F	A2h1	12	1	DPGN IV/Mem V
20–13	12	14	2	F	D1c	10	2	DPGN IV
20–15	16	17	1	F	A2o	6	0	DPGN IV
20–21	7	23	17	F	A2w	8	16	DPGN IV/CKD
20–25	8	12	4	F	C1c*4	6	3	DPGN IV
20–34	11	12	0.5	F	B2d	12	0	No bx
21–37	2	18	16	F	A2	2	0	DPGN IV
21–39	8	9	1	F	A2	29	0	DPGN IV
21–57	15	16	1	F	C1b	8	0	DPGN IV
Caucasian								
20–01	17	17	0.25	F	H1aq1	6	0	No bx
20–17	10	16	6	F	U5a1d1	0	0	No bx
20–32	4	12	8	F	H3	8	0	DPGN IV
20–35	12	16	4	F	T2b21	5	0	Mes II/Mem V
Asian								
20–29	8	20	12	M	B4c1b2a	4	0	Mes II/Mem V
21–44	14	19	5	F	N	4	0	Mes II/Mem V
21–53	12	20	8	F	D4	2	0	No bx
21–59	5	9	5	F	D5c2	2	0	No bx

**Table 2:** Demographic differences between haplogroups.

	African n = 5	Amerindian n = 13	Caucasian n = 4	Asian n = 4	Average %	*p*
^1^ Sex, n (%)						0.99
Female	4 (80%)	11 (85%)	4 (100%)	3 (75%)	85%	
Male	1 (20%)	2 (15%)		1 (25%)	15%	
^2^ Current Age (years), Mean (SD)	15.0 (5.2)	15.5 (3.8)	15.2 (2.2)	17 (5.4)	NA	0.90
^2^ Age at Diagnosis (years), Mean (SD)	10.0 (4.7)	10.8 (4.1)	10.8 (5.4)	9.8 (4.0)	NA	0.97
^2^ SLE Duration (years), Mean (SD)	5.0 (6.0)	4.9 (5.5)	4.6 (3.3)	7.5 (3.3)	NA	0.82

^1^ Exact Test. ^2^ Analysis of Variance. NA—Not applicable.

**Table 3:** SLEDAI-2K and SDI differences between haplogroups.

	African n = 5	Amerindian n = 13	Caucasian n = 4	Asian n = 4	Omnibus *p* ^1^	African vs. Amerindian *p* ^2^	African vs. Caucasian *p* ^2^	African vs. Asian *p* ^2^	Amerindian vs. Caucasian *p* ^2^	Amerindian vs. Asian *p* ^2^	Caucasian vs. Asian *p* ^2^
SLEDAI-2K, Median [25th, 75th]	8 [8, 8]	10 [6, 14]	5.5 [2.5, 7]	3 [2, 4]	0.017	0.293	0.159	0.074	0.045	0.008	0.242
SDI, Median [25th, 75th]	0 [0, 2]	1 [0, 2]	0 [0, 0]	0 [0, 0]	0.121	0.672	0.179	0.179	0.075	0.075	NE
Non-Zero SDI, n (%)	2 (40%)	7 (54%)	0 (0%)	0 (0%)	0.103	1.000	0.444	0.444	0.102	0.102	NE

^1^ Kruskal–Wallis Test or Exact Test (for Non-Zero SDI). ^2^ Wilcoxon Rank-Sum Test or Exact Test (for non-zero SDI). NE = not estimable.

**Table 4:** Disease duration and activity within the haplogroups.

	Average Age (Years)	Disease Duration	SLEDAI > 6	SDI > 1
Amerindian (n = 13)	15.5	4.9	92% *	54% ^†^
African (n = 5)	15.0	5.0	80% *	40%
Caucasian (n = 4)	15.3	4.6	50%	0%
Asian (n = 4)	17.0	7.5	0%	0%

* *p* < 0.05 for comparison to Asian haplogroup using Fisher’s exact test. ^†^ *p* = 0.10 for comparison to Caucasian and Asian haplogroups using Fisher’s exact test.

**Table 5:** Incidence and classification of lupus nephritis by lineage.

	Lupus Nephritis Disease Class II	Lupus Nephritis Class III/IV	Lupus Nephritis Class V	CKD/ESRD
Amerindian (n = 13)	0%	85%	7%	15%
African (n = 5)	0%	80%	0%	20%
Caucasian (n = 4)	25%	50%	25%	0%
Asian (n = 4)	50%	0%	50%	0%

* *p* < 0.05 for comparison to Asian haplogroup using Fisher’s exact test.

## Data Availability

The original contributions presented in this study are included in the article. Further inquiries can be directed to the corresponding author(s).

## Supplementary Materials

The following supporting information can be downloaded at https://www.mdpi.com/article/10.3390/jcm15010086/s1, Table S1: The 1997 ACR Criteria for the diagnosis of SLE. Table S2: SLEDAI-2K disease activity scores. Table S3: Systemic Lupus International Collaborating Clinics/American College of Rheumatology Damage Index (SDI). Table S4: SLEDAI scores for each patient. Table S5: Haplogroup and maternal ancestral origin group risk based on SLEDAI scores. Table S6: SDI scores for each patient. Table S7: Haplogroup and maternal ancestral origin group risk based on SDI scores.
