# Supplementary material for: Association of Mitochondrial DNA Haplogroups with Pediatric Systemic Lupus Erythematosus Disease Activity, Damage Scores, and Lupus Nephritis
Source: J Clin Med. 2025 Dec 23;15(1):86. doi: 10.3390/jcm15010086 (PMC12786546; doi:10.3390/jcm15010086)
Supplement: Supplementary file 1 [file jcm-15-00086-s001.zip › jcm-3957859-supplementary.pdf]

Supplementary Materials:

Table S1: 1997 ACR Criteria for Diagnosis of SLE

Malar rash over the bridge of nose and cheekbones, sparing the nasal labial fold, only involves the epithelium, thus non-scarring
Discoid Rash that is well circumscribed and involves the dermis and often scars.
Photosensitivity
Oral or nasal ulcers
Arthritis defined as inflammation in 2 or more peripheral joints
Serositis involvening the pleura or pericardium
Renal invovlement with proteinuria > 500 mg/24 hours or cellular casts
Neurologic disorder including seizures, psychosis or delirium.
Hematologic disorder including autoimmune hemalytic anemia, thrombocytopenia, or leukopenia
Immunologic disorder includin a positive anti-dsDNA, anti-Sm, or anti-phospholipid antibodies
Positive antinuclear antibody (ANA)

Total	

Must have 4/11 criteria ro make a diagnosis of SLE. The Table made from data in References 1-3.

Table S2 SLEDAI-2K Disease Activity Score

Recent onset seizure<br>Exclude metabolic, infectious, or drug causes	If positive 8 points
Psychosis<br>Altered ability to function in normal activity due to severe disturbance in the perception of reality (include hallucinations, incoherence, marked loose associations, impoverished thought content, marked illogical thinking, and bizarre, disorganized, or catatonic behavior); exclude uremia and drug causes	If positive 8 points
Organic brain syndrome<br>Altered mental function with impaired orientation, memory, or other intellectual function (with rapid onset and fluctuating clinical features), inability to sustain attention to environment, and ≥2 of the following: perceptual disturbance, incoherent speech, insomnia or daytime drowsiness, and increased or decreased psychomotor activity; exclude metabolic, infectious, or drug causes	If positive 8 points
Visual disturbance<br>Retinal changes of SLE (include cytoid bodies, retinal hemorrhages, serous exudates or hemorrhages in choroid, and optic neuritis); exclude hypertensive, infectious, or drug causes	If positive 8 points
New onset sensory or motor neuropathy involing cranial nerves	If positive 8 points
Lupus headache<br>Severe, persistent headache (may be migrainous but must be nonresponsive to narcotic analgesia)	If positive 8 points

New onset stroke<br>Exclude arteriosclerosis	If positive 8 points
Vasculitis<br>Ulceration, gangrene, tender finger nodules, periungual infarction, splinter hemorrhages or biopsy, and angiogram proof of vasculitis	If positive 8 points
Arthritis<br>≥2 joints with pain and signs of inflammation (i.e., tenderness, swelling, or effusion)	If positive 4 points
Myositis<br>Proximal muscle aching/weakness associated with<br>elevated CPK/aldolase, EMG changes, or a biopsy showing myositis	If positive 4 poitns
Heme-granular or RBC urinary casts	If positive 4 points
Hematuria<br>>5 RBC/high-power field; exclude stone, infection, or other cause	If positive 4 points
Proteinuria<br>>0.5 g/24 hours	If positive 4 points
Pyuria<br>>5 WBC/high-power field; exclude infection	If positive 4 points
Inflammatory-type rash	If positive 2 points
Alopecia	If positive 2 points
Oral or nasal mucosal ulcers	If positive 2 points
Pleuritic chest pain with pleural rub/effusion or pleural thickening	If positive 2 points
Low complement<br>CH50, C3, or C4 decreased below lower limit of normal for lab	If positive 2 points

High DNA binding<br>Increased above normal range for lab	If positive 2 points
Temp >100.4 °F (38°C)<br>Exclude infectious causes	If positive 1 point
Platelets <100 x 10^9/L<br>Exclude drug causes	If positive 1 point
WBC <3 x 10^9/L<br>Exclude drug causes	If positive 1 point
Total	

A score of 4 or greater usually indicate the need for increased therapy. In our patient cohort we used a acore of 6 or greater to indicate active disease.
The chart was made from information gathered from Reference 3.

Table S3 Systemic Lupus International Collaborating Clinics/American College Of Rheumatology Damage Index (SDI)

Ocular: Cataract or Retinal changes/retinal atrophy
Neuropsychiatric: Cognitive impairment or major psychosis, Seizures for > 6 months, Cerebral vascular accident, Cranial or peripheral neuropathy
Renal: Glomerular filtration rate (GFR) <50%, Proteinuria > 3.5 gm/24 hours, End stage renal disease
Pulmonary: Pulmonary hypertension, Pulmonary fibrosis, Shrinking lung syndrome, Pleural fibrosis, Pulmonary Infarct
Cardiovascular: Angina or coronary bypass, Myocardial infardct, Crdiomyopathy, Valvular Disease, Pericarditis 6 months or more, Pericardectomy
Periphearl Vascular: Claudication for 6 months, Minor tissue loss (pulp space), Major tissue loss, Venous thrombosis
Gastrointestinal: Infarct or resection of bowel, spleen, liver, gall bladder, Mesenteric insufficiency, Chronic peritonitis, Sricture of GI tract, Pancreatic insufficiency
Musculoskeletal: Muscle atrophy or weakness, Deforming/erosive arthritis, Osteroporosis, Avascular necorosis, Osteromyelitis, Tendon rupture
Skin: Scarring alopecia, extensive scarring or panniculum, Skin ulceration . 6 months
Diabetes:
Premature Gonadal Failure:
Malignancy:
Total

Each of these condition is consider more permanent damage and can range from a score of 1-2.
The chart was made from information gathered from Reference 6

A score

Sample	Self-identify	Haplo group	*4.Visual Disturbance	*8.Vas culitis	*9.Art hritis	*12.Hem aturia	*13.P yuria	*14.Prot einuria	*15. Rash	*18.Peri carditis	*19.Low Complement	*20.Increased DNA Binding	Total
**SLE-20-01**	Afri	H	0	0	1	0	0	0	0	0	0	1	2/24
**SLE-20-03**	Hisp	L	0	0	0	0	0	0	0	0	0	0	0/24
**SLE-20-05**	Hisp	A	0	0	0	0	0	0	0	0	0	0	0/24
**SLE-20-07**	Hisp	A	0	0	0	0	0	0	0	0	0	0	0/24
**SLE-20-09**	Hisp	A	0	0	0	0	0	0	0	0	0	0	0/24
**SLE-20-11**	Hisp	A	0	0	0	1	0	1	1	0	1	0	4/24
**SLE-20-13**	Hisp	D	0	0	0	1	0	1	0	1	0	0	3/24
**SLE-20-15**	Hisp	L	0	0	1	0	0	0	0	0	0	1	2/24
**SLE-20-16**	Afri	A	0	0	0	1	0	1	0	0	0	1	3/24
**SLE-20-17**	Euro	U	0	0	0	0	0	0	0	0	0	0	0/24
**SLE-20-19**	Afri	L	0	0	0	1	0	0	0	0	1	1	3/24
**SLE-20-21**	Hisp	A	1	0	0	0	1	1	0	0	0	0	3/24
**SLE-20-23**	Afri	L	0	0	0	0	0	0	0	0	0	1	1/24
**SLE-20-25**	Hisp	C	0	0	0	1	0	1	0	0	0	0	2/24
**SLE-20-32**	Hisp	H	0	0	0	1	0	1	1	0	1	0	4/24
**SLE-20-34**	Hisp	B2	0	1	0	0	0	0	0	0	1	1	3/24
**SLE-21-35**	Hisp	T	0	0	0	0	0	0	0	0	0	0	0/24
**SLE-21-37**	Hisp	A	0	0	0	0	0	0	0	0	0	0	0/24
**SLE-21-39**	Hisp	A	0	0	0	0	0	0	0	0	0	0	0/24
**SLE-21-46**	Hisp	M	0	0	0	0	0	0	0	0	1	0	1/24
**SLE-21-53**	Asia	M	0	0	0	0	0	0	0	0	0	1	1/24
**SLE-21-55**	Hisp	L	0	0	1	0	0	0	0	0	1	1	3/24
**SLE-21-57**	Hisp	M	0	0	1	0	0	0	0	0	1	1	3/24
**SLE-21-59**	Asia	M	0	0	0	0	0	0	0	0	0	0	0/24
**SLE-20-29**	Asia	B4	0	0	0	0	0	0	0	0	1	1	2/24
**Total symptoms**			1	1	4	6	1	6	2	1	8	10	

*Legend: *Only showing symptoms reported by patients. African-Afri; Hispanic-Hisp; European-Euro;Asian-Asia*

1.Seizure; 2.Psychosis; 3.Organic Brain Syndrome; 4.Visual Disturbance; 5.Cranial Nerve Disorder; 6.Lupus Headache; 7.CVA; 8.Vasculitis; 9.Arthritis; 10.Myositis; 11.Urinary casts; 12.Hematuria; 13.Pyuria; 14.Proteinuria; 15.Rash; 16.Mucosal Ulcers; 17.Pleurisy; 18.Pericarditis; 19.Low Complement; 20.Increased DNA Binding; 21.Fever; 22.Thrombocytopenia; 23.Leukopenia; 24.Alopecia

Table S5 Haplogroup and maternal ancestral origin group risk based on SLEDAI scores

**SLEDAI Symptoms #**		**9.Arthritis**	**12.Hematuria**	**14.Proteinuria**	**19.Low Complement**	**20.Increased DNA Binding**
**Total number of patients with symptoms**		4	6	6	8	10
**Maternal Ancestral Origin**	**Haplogroup**					
**African**	**L**	2 (50.0%)	1 (16.7%)	0 (0.0%)	2 (25.0%)	4 (40.0%)
**Asian**	**M,B4**	1 (25.0%)	0 (0.0%)	0 (0.0%)	3 (37.5%)	3 (30.0%)
**AmerIndian**	**A,B2,C,D**	0 (0.0%)	4 (66.7%)	5 (83.3%)	2 (25.0%)	2 (20.0%)
**European**	**H,T,U**	1 (25.0%)	1 (16.7%)	1 (16.7%)	1 (12.5%)	1 (10.0%)

Table S6 SDI scores for each patient

Sample	Self-identify	Haplo group	*1.Retinal…	*2.Cataract	*8GFR <50%	*9.Proteinuria…	*10.End-stage…	*11.Pulmonary…	*14.Pleural…	*19.Valvular…	*22.Minor Tissue Loss…	*26.Mesenteric…	Total
**SLE-20-01**	Afri	H	0	0	0	0	0	0	0	0	0	0	0/39
**SLE-20-03**	Hisp	L	0	0	0	0	0	0	0	0	0	0	0/39
**SLE-20-05**	Hisp	A	0	0	0	0	0	0	0	0	0	0	0/39
**SLE-20-07**	Hisp	A	0	0	0	0	0	0	0	0	0	0	0/39
**SLE-20-09**	Hisp	A	0	0	0	0	0	0	0	0	0	0	0/39
**SLE-20-11**	Hisp	A	0	0	1	0	0	0	0	0	0	0	1/39
**SLE-20-13**	Hisp	D	0	0	1	0	0	0	0	1	0	0	2/39
**SLE-20-15**	Hisp	L	0	0	0	0	0	0	0	0	0	0	0/39
**SLE-20-16**	Afri	A	0	0	0	1	0	0	0	0	0	0	1/39
**SLE-20-17**	Euro	U	0	0	0	0	0	0	0	0	0	0	0/39
**SLE-20-19**	Afri	L	0	0	0	0	0	0	0	0	0	0	0/39
**SLE-20-21**	Hisp	A	1	1	1	0	1	1	0	0	1	1	7/39
**SLE-20-23**	Afri	L	0	0	0	0	0	0	0	0	0	0	0/39
**SLE-20-25**	Hisp	C	0	1	1	0	0	0	1	0	0	0	3/39
**SLE-20-32**	Hisp	H	0	0	0	0	0	0	0	0	0	0	0/39
**SLE-20-34**	Hisp	B2	0	0	0	0	0	0	0	0	0	0	0/39
**SLE-21-35**	Hisp	T	0	0	0	0	0	0	0	0	0	0	0/39
**SLE-21-37**	Hisp	A	0	0	0	0	0	0	0	0	0	0	0/39
**SLE-21-39**	Hisp	A	0	0	0	0	0	0	0	0	0	0	0/39
**SLE-21-46**	Hisp	M	0	0	0	0	0	0	0	0	0	0	0/39
**SLE-21-53**	Asia	M	0	0	0	0	0	0	0	0	0	0	0/39
**SLE-21-55**	Hisp	L	0	0	0	0	0	0	0	0	0	0	0/39
**SLE-21-57**	Hisp	M	0	0	0	0	0	0	0	0	0	0	0/39
**SLE-21-59**	Asia	M	0	0	0	0	0	0	0	0	0	0	0/39
**SLE-20-29**	Asia	B4	0	0	0	0	0	0	0	0	0	0	0/39
**Total symptoms**			1	2	4	1	1	1	1	1	1	1	

*Legend: *Only showing symptoms reported by patients. African-Afri; Hispanic-Hisp; European-Euro;Asian-Asia*
1.Retinal change or optic atrophy; 2.Cataract; 3.Cognitive Impairment or Major Psychosis; 4.Seizures requiring therapy for ≥6 months; 5.Cerebrovascular Accident; 6.Cranial or Peripheral Neuropathy; 7.Transverse Myelitis; 8.Estimated or Measured GFR <50%; 9.Proteinuria ≥3.5 g/24 hours; 10.End-stage renal disease; 11.Pulmonary Hypertension;

12.Pulmonary Fibrosis; 13.Shrinking Lung; 14.Pleural Fibrosis; 15.Pulmonary Infarction; 16.Angina or Coronary Artery Bypass; 17.Myocardial Infarction; 18.Cardiomyopathy; 19.Valvular disease; 20.Pericarditis or Pericardiectomy; 21.Claudication; 22.Minor Tissue Loss from Peripheral Vascular Disease; 23.Significant Tissue Loss from Peripheral Vascular Disease; 24.Venous Thrombosis with Swelling, Ulceration or Venous Stasis; 25.Infarction or Resection of Bowel (below duodenum), Spleen, Liver or Gallbladder; 26.Mesenteric Insufficiency; 27.Chronic Peritonitis; 28.Stricture or Upper Gastrointestinal Tract Surgery; 29.Pancreatic Insufficiency Requiring Enzyme Replacement or with Pseudocyst; 30.Muscle Atrophy or Weakness; 31.Deforming or Erosive Arthritis; 32.Osteoporosis with Fracture or Vertebral Collapse; 33.Avascular Necrosis; 34.Scarring Chronic Alopecia; 35.Extensive Scarring of Panniculus other than Scalp and Pulp Space; 36.Skin Ulceration (excluding thrombosis); 37.Premature Gonadal Failure; 38.Diabetes Requiring Therapy; 39.Malignancy

Table S7 Haplogroup and maternal ancestral origin group risk based on SDI scores

**SDI Symptoms #**	**8.Estimated or Measured GFR <50%**	
**Total number of patients with symptoms**	4	
**Maternal Ancestral Origin**	**Haplogroup**	25
**African**	**L**	0 (0.0%)
**Asian**	**M,B4**	0 (0.0%)
**AmerIndian**	**A,B2,C,D**	4 (100.0%)
**European**	**H,T,U**	0 (0.0%)
